# Salivary lipocalin family proteins from *Panstrongylus chinai*, a vector of Chagas disease

**DOI:** 10.1016/j.dib.2017.09.039

**Published:** 2017-09-22

**Authors:** Hirotomo Kato, Ryan C. Jochim, Eduardo A. Gomez, Shunsuke Tsunekawa, Jesus G. Valenzuela, Yoshihisa Hashiguchi

**Affiliations:** a Division of Medical Zoology, Department of Infection and Immunity, Jichi Medical University, Shimotsuke, Tochigi, Japan; b Vector Molecular Biology Section, Laboratory of Malaria and Vector Research, National Institute of Allergy and Infectious Diseases, NIH, Rockville, MD, USA; c Departamento de Parasitologia y Medicina Tropical, Centro de Biomedicina, Facultad de Ciencias Medicas, Universidad Catolica de Santiago de Guayaquil, Guayaquil, Ecuador; d Laboratory of Parasitology, Department of Disease Control, Graduate School of Veterinary Medicine, Hokkaido University, Sapporo, Hokkaido, Japan

**Keywords:** *Panstrongylus chinai*, Saliva, Lipocalin, Transcriptome

## Abstract

The dataset in this report is related to the research article with the title: “Salivary gland transcripts of the kissing bug, *Panstrongylus chinai*, a vector of Chagas disease” (Kato et al., 2017) [1]. Lipocalin family proteins were identified as the dominant component in *P. chinai* saliva, and phylogenetic analysis of the salivary lipocalins resulted in the formation of five major clades. For further characterization, each clade of *P. chinai* lipocalin was s alignment and phylogenetic analyses together with homologous triatomine lipocalins; pallidipin 2, an inhibitor of collagen-induced platelet aggregation identified from saliva of *Triatoma pallidipennis* (clade I), pallidipin-like salivary lipocalin from *Triatoma dimidiata* (clade II), salivary lipocalin from *T. dimidiata* (clade III), triatin-like salivary lipocalin identified in the saliva of *T. dimidiata* (clade IV), and lipocalin-like TiLipo37 from *Triatoma infestans* (clade V).

**Specifications Table**

**Table:** 

Subject area	*Biology*
More specific subject area	*Salivary lipocalins of a hematophagous insect*
Type of data	*Table, figure*
How data was acquired	*Transcriptome, alignment and phylogenetic analyses*
Data format	*Analyzed*
Experimental factors	*A dataset of transcripts from salivary grands of Panstrongylus chinai*
Experimental features	*Alignment and phylogenetic analyses of salivary lipocalins from Panstrongylus chinai*
Data source location	*Ecuador*
Data accessibility	*Accession numbers of the sequence data are available in the reference**[1]**.*

**Value of the data**

• The data is the second report of the salivary lipocalins in a *Panstrongylus* species.
• The result will provide further information into the salivary biochemical and pharmacological complexity of triatomine bugs and the evolution of salivary components in blood sucking arthropods.
• cDNAs and recombinant proteins prepared from these transcripts will result in the discovery of novel pharmacologically active compounds, as well as the development of biomarkers following exposure to *Panstrongylus chinai*.

## 1 Data

**Fig. 1:**
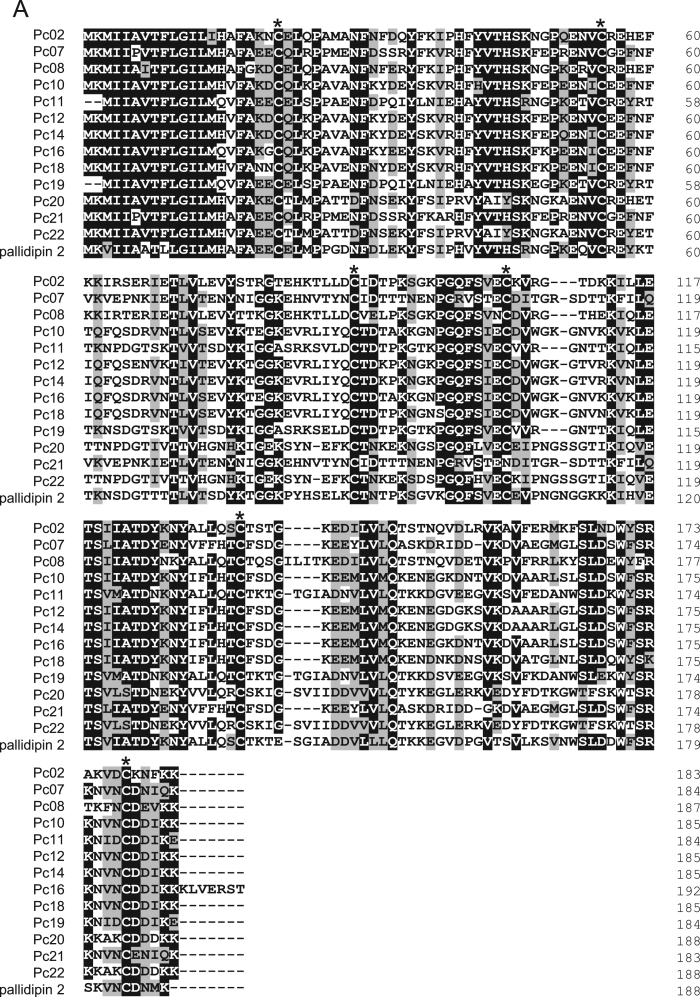

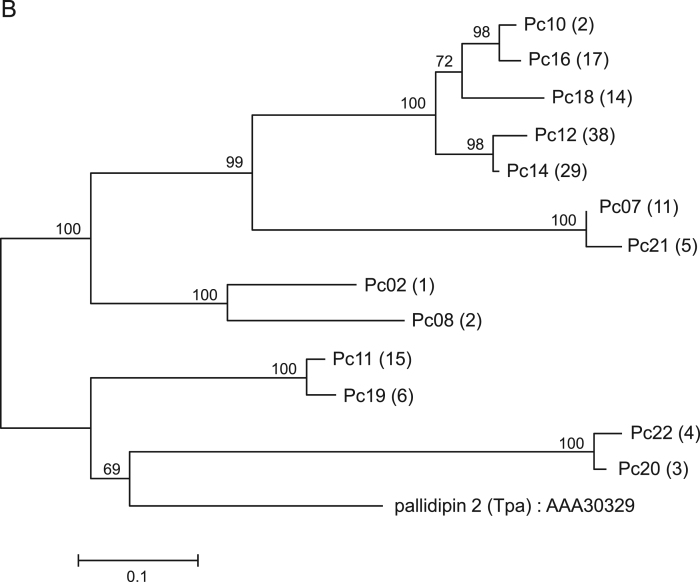
(A) Sequence alignment of pallidipin 2-like proteins from *Panstrongylus chinai* (Pc02, Pc07, Pc08, Pc10-Pc12, Pc14, Pc16 and Pc18-Pc22) together with pallidipin 2 from *Triatoma pallidipennis* (accession number: AAA30329). Black-shaded amino acids represent identical amino acids and gray-shaded amino acids represent conserved amino acids. Dashes indicate gaps introduced for maximal alignment. Asterisks at the top of the amino acids denote conserved cysteine residues. (B) Phylogenetic tree analysis of pallidipin 2-like proteins (Pc02, Pc07, Pc08, Pc10-Pc12, Pc14, Pc16 and Pc18-Pc22) from *P. chinai* with pallidipin 2 from *T. pallidipennis*. The numbers in parentheses indicate the number of transcripts of each contig. The scale bar represents 0.1% divergence. Bootstrap values are shown above branches.

**Fig. 2:**
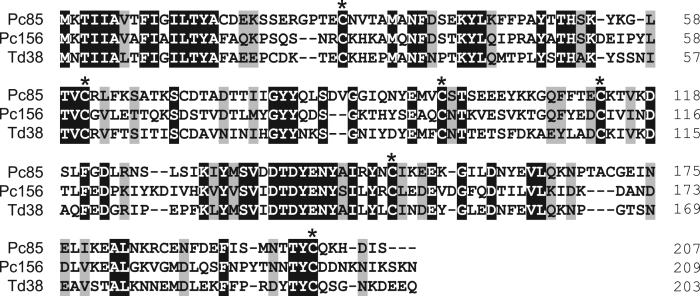
Sequence alignment of Td38-like proteins from *Panstrongylus chinai* (Pc85 and Pc156) together with Td38 from *Triatoma dimidiata* (accession number: AB470389). Black-shaded amino acids represent identical amino acids and gray-shaded amino acids represent conserved amino acids. Dashes indicate gaps introduced for maximal alignment. Asterisks at the top of the amino acids denote conserved cysteine residues.

**Fig. 3:**
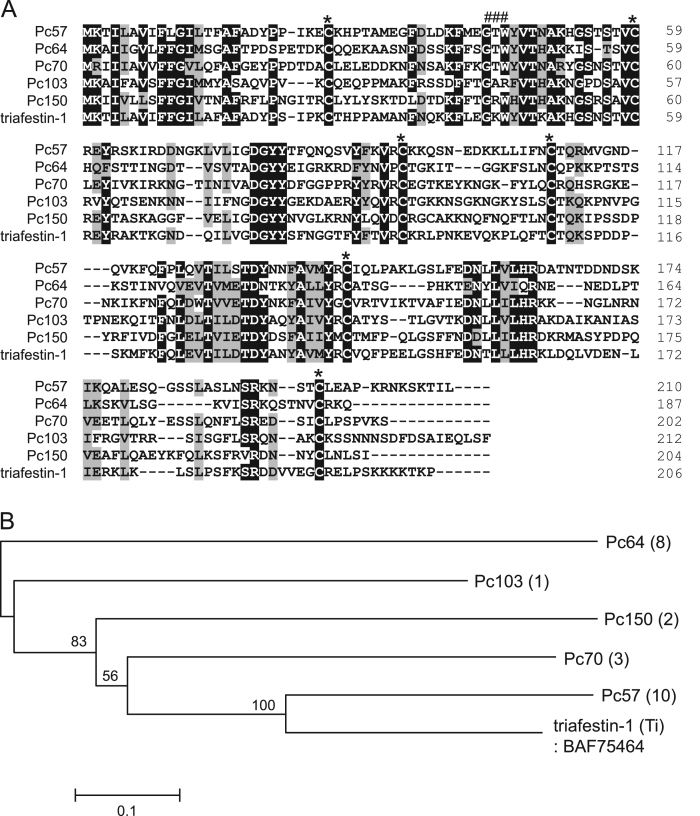
(A) Sequence alignment of triafestin-1-like proteins from *Panstrongylus chinai* (Pc57, Pc64, Pc70, Pc103 and Pc150) together with triafestin-1 from *Triatoma infestans* (accession number: BAF75464). Black-shaded amino acids represent identical amino acids and gray-shaded amino acids represent conserved amino acids. Dashes indicate gaps introduced for maximal alignment. Asterisks at the top of the amino acids denote conserved cysteine residues, and the GXW motif is indicated by ###. (B) Phylogenetic tree analysis of triafestin-1-like proteins from *P. chinai* (Pc57, Pc64, Pc70, Pc103 and Pc150) with triafestin-1 from *T. infestans*. The numbers in parentheses indicate the number of transcripts of each contig. The scale bar represents 0.1% divergence. Bootstrap values are shown above branches.

**Fig. 4:**
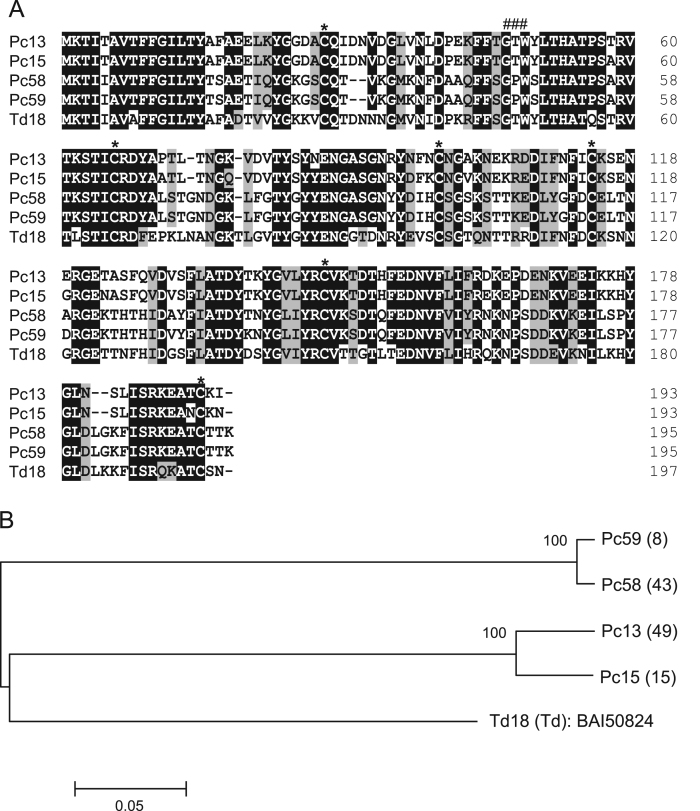
(A) Sequence alignment of Td18-like proteins from *Panstrongylus chinai* (Pc13, Pc15, Pc58 and Pc59) together with Td18 from *Triatoma dimidiata* (accession number: BAI50824). Black-shaded amino acids represent identical amino acids and gray-shaded amino acids represent conserved amino acids. Dashes indicate gaps introduced for maximal alignment. Asterisks at the top of the amino acids denote conserved cysteine residues, and the GXW motif is indicated by ###. (B) Phylogenetic tree analysis of Td18-like proteins from *P. chinai* (Pc13, Pc15, Pc58 and Pc59) together with Td18 from *T. dimidiata*. The numbers in parentheses indicate the number of transcripts of each contig. The scale bar represents 0.05% divergence. Bootstrap values are shown above branches.

**Fig. 5:**
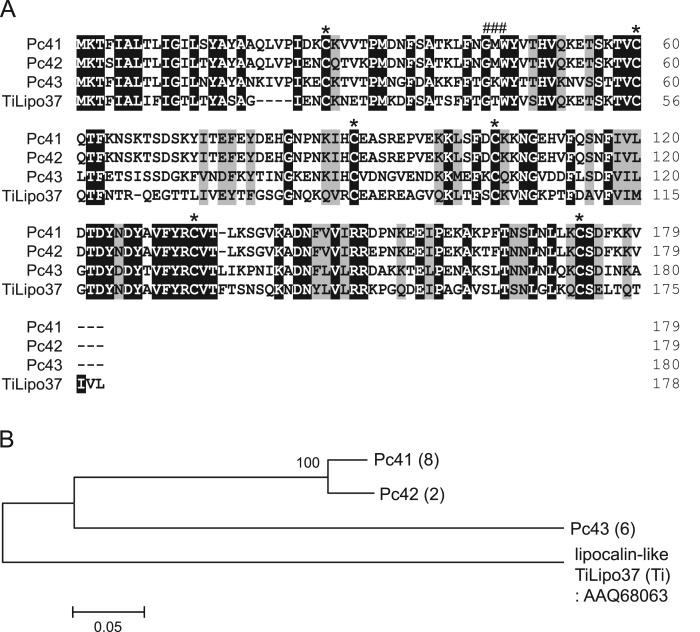
(A) Sequence alignment of lipocalin-like TiLipo37-like proteins from *Panstrongylus chinai* (Pc41-Pc43) together with lipocalin-like TiLipo37 from *Triatoma infestans* (accession number: AAQ68063). Black-shaded amino acids represent identical amino acids and gray-shaded amino acids represent conserved amino acids. Dashes indicate gaps introduced for maximal alignment. Asterisks at the top of the amino acids denote conserved cysteine residues, and the GXW motif is indicated by ###. (B) Phylogenetic tree analysis of lipocalin-like TiLipo37-like proteins from *P. chinai* (Pc41-Pc43) together with lipocalin-like TiLipo37 from *T. infestans*. The numbers in parentheses indicate the number of transcripts of each contig. The scale bar represents 0.05% divergence. Bootstrap values are shown above branches.

The dominant transcripts of *Panstrongylus chinai* salivary glands were analyzed by sequence analysis of the cDNA library, and 73.7% of transcripts encoding the putative secreted proteins coded for the lipocalin family of proteins [1]. Table 1 shows the grouping of transcripts coding for lipocalin family proteins in *P. chinai* salivary glands obtained by the phylogenetic analysis [1]. Fig. 1, Fig. 2, Fig. 3, Fig. 4, Fig. 5 represent alignment and phylogenetic analyses of each clade of *P. chinai* salivary lipocalins together with homologous proteins; pallidipin 2, a platelet aggregation inhibitor identified from *Triatoma pallidipennis* saliva (clade I), pallidipin-like salivary lipocalin from *Triatoma dimidiata* saliva with unknown function (clade II), salivary lipocalin from *T. dimidiata* saliva with unknown function (clade III), triatin-like salivary lipocalin identified in the saliva of *T. dimidiata* with unknown function (clade IV), and lipocalin-like TiLipo37 from *Triatoma infestans* saliva with unknown function (clade V), showing their structural similarity and diversity.

**Table 1:** Transcripts coding for lipocalin family proteins in *Panstrongylus chinai* salivary glands.

Clade	Similar to	No. of clusters	No. of seq	% seq
Clade I				
	pallidipin 2 (*Triatoma pallidipennis*): AAA30329	16	151	46.9
	Td42, similar to pallidipin 2 (*Triatoma dimidiata*): BAI50842	1	1	0.3
Clade II				
	Td38, similar to pallidipin-like salivary lipocalin (*Triatoma dimidiata*): BAI50839	2	5	1.6
	Td33, similar to pallidipin-like salivary lipocalin (*Triatoma dimidiata*): BAI50837	1	1	0.3
Clade III				
	Td26, similar to salivary lipocalin (*Triatoma dimidiata*): BAI50831	2	11	3.4
	salivary lipocalin (*Triatoma infestans*): ABR27920	1	8	2.4
	Td40, similar to triabin-like lipocalin 4a precursor (*Triatoma dimidiata*): BAI50840	1	3	0.9
	triabin-like lipocalin 4a precursor (*Triatoma infestans*): ABR27959	1	2	0.7
	salivary lipocalin (*Triatoma infestans*): ABR27868	1	1	0.3
Clade IV				
	Td18, similar to triatin-like salivary lipocalin (*Triatoma dimidiata*): BAI50824	4	66	20.5
	Td11, similar to triatin-like salivary lipocalin (*Triatoma dimidiata*): BAI50818	1	43	13.3
	Td45, similar to pallidipin-like salivary lipocalin (*Triatoma dimidiata*): BAI50844	1	8	2.5
Clade V				
	lipocalin-like TiLipo37 (*Triatoma infestans*): AAQ68063	2	10	3.1
	salivary lipocalin (*Triatoma brasiliensis*): ABH09436	1	6	1.9
Others				
	Td24, similar to salivary lipocalin (*Triatoma dimidiata*): BAI50829	2	2	0.7
	triabin-like salivary lipocalin (*Triatoma infestans*): ABR27927	1	1	0.3
	salivary lipocalin (*Triatoma infestans*): ABR27831	1	1	0.3
	salivary lipocalin 1 (*Triatoma brasiliensis*): ABH09421	1	1	0.3
	Td23, similar to salivary lipocalin (*Triatoma dimidiata*): BAI50828	1	1	0.3
Total		41	322	100.0

## 2 Experimental design, materials and methods

The sequences of *P. chinai* salivary lipocalins were obtained in the study “Salivary gland transcripts of the kissing bug, *Panstrongylus chinai*, a vector of Chagas disease” [1]. The sequences coding for lipocalin family of proteins by BLASTx analyses were aligned with CLUSTAL W software [2] and examined using Molecular Evolutionary Genetics Analysis (MEGA) version 5.2 [3]. Phylogenetic trees by the neighbor-joining method were constructed with the distance algorithms available in the MEGA package. Bootstrap values were determined on 1000 replicates of the data sets. Accession numbers of the sequence data are available in “Salivary gland transcripts of the kissing bug, *Panstrongylus chinai*, a vector of Chagas disease” [1].
